# The Thiamine diphosphate dependent Enzyme Engineering Database: A tool for the systematic analysis of sequence and structure relations

**DOI:** 10.1186/1471-2091-11-9

**Published:** 2010-02-01

**Authors:** Michael Widmann, Robert Radloff, Jürgen Pleiss

**Affiliations:** 1 Institute of Technical Biochemistry, University of Stuttgart, Allmandring 31, 70569 Stuttgart, Germany

## Abstract

**Background:**

Thiamine diphosphate (ThDP)-dependent enzymes form a vast and diverse class of proteins, catalyzing a wide variety of enzymatic reactions including the formation or cleavage of carbon-sulfur, carbon-oxygen, carbon-nitrogen, and especially carbon-carbon bonds. Although very diverse in sequence and domain organisation, they share two common protein domains, the pyrophosphate (PP) and the pyrimidine (PYR) domain. For the comprehensive and systematic comparison of protein sequences and structures the Thiamine diphosphate (ThDP)-dependent Enzyme Engineering Database (TEED) was established.

**Description:**

The TEED http://www.teed.uni-stuttgart.de contains 12048 sequence entries which were assigned to 9443 different proteins and 379 structure entries. Proteins were assigned to 8 different superfamilies and 63 homologous protein families. For each family, the TEED offers multisequence alignments, phylogenetic trees, and family-specific HMM profiles. The conserved pyrophosphate (PP) and pyrimidine (PYR) domains have been annotated, which allows the analysis of sequence similarities for a broad variety of proteins. Human ThDP-dependent enzymes are known to be involved in many diseases. 20 different proteins and over 40 single nucleotide polymorphisms (SNPs) of human ThDP-dependent enzymes were identified in the TEED.

**Conclusions:**

The online accessible version of the TEED has been designed to serve as a navigation and analysis tool for the large and diverse family of ThDP-dependent enzymes.

## Background

Since the discovery of the first thiamine diphosphate (ThDP)-dependent enzyme in 1937, a multitude of them has been described and their catalytic mechanism was intensively analysed [1-3]. ThDP-dependent enzymes catalyze a wide variety of enzymatic reactions and therefore were assigned to the families of oxidoreductases, transferases, or lyases [4]. The formation or cleavage of carbon-sulfur, carbon-oxygen, carbon-nitrogen, and especially carbon-carbon bonds are of utmost interest for bioorganic synthesis and organocatalysis [5,6]. Because of their ability to form asymmetric C-C bonds, ThDP-dependent enzymes are versatile catalysts for a variety of biotransformations [7-12]. In addition, the ThDP-dependent enzyme family has been shown to possess a wide substrate spectrum ranging from small compounds like formaldehyde to bulky hydroxyl-phytanoyl-CoA molecules [13,14]. For pharmacology, ThDP-dependent enzymes of human origin are of special interest. They have been identified as being involved in a variety of diseases like Alzheimer's disease and diabetes [15], and also play a role in tumor proliferation [16]. Their highly diverse substrate specificity and catalytic activity is reflected in their sequence and structure which differs significantly between different families of ThDP-dependent enzymes. During the course of evolution, shuffling, rearrangement, and fusion of domains, as well as mutation, and gene duplications have led to the enormous diversity of ThDP-dependent enzymes [17,18]. However, all ThDP-dependent enzymes contain at least two conserved domains, the pyrophosphate (PP) and the pyrimidine (PYR) domain, which have a similar structure [18] and are essential for binding and activating ThDP [19]. The PYR domain has a conserved catalytic glutamic acid while the PP domain contains a conserved GDX_25-30_N motif [17,20-22]. In addition to these two domains, additional domains were found such as the the transhydrogenase dIII domain (TH3) and the transketolase C-terminal domain (TKC) [17,18,23]. These additional domains are often not well characterised and in many cases their function in the catalytic process remains obscure [17]. A unified classification scheme for ThDP-dependent enzymes based on a comprehensive analysis of sequence and structure does not yet exist. Based on a structural comparison, it was suggested that a total of 4 families should be sufficient to describe ThDP-dependent enzymes: DC (decarboxylases), TK (transketolases), OR (oxidoreductases), and KD (2-ketoacid dehydrogenase) [18]. A sequence based evolutionary analysis suggested at least 6 different families, namely TK (transketolases)-like, PFRD (pyruvate ferredoxin reductase), 2OXO (2-oxoisovalerate dehydrogenase)-like, PDC (pyruvate decarboxylase)-like, SPDC (sulfopyruvate decarboxylase), and PPDC (phosphopyruvate decarboxylase) [17].

We established the Thiamine diphosphate dependent Enzyme Engineering Database (TEED) as a tool for a comprehensive and systematic comparison of ThDP-dependent enzymes from different protein families and annotated the conserved PP- and PYR domains. Thus, the TEED is the first data resource of ThDP-dependent enzymes which combines information on the individual protein families, sequence alignments and a consistent annotation of the conserved PYR and PP domains.

## Construction and content

### Source Data

The Thiamine diphosphate (ThDP)-dependent Enzyme Engineering Database (TEED) was established by utilising the data warehouse system DWARF [24]. The DWARF system is a collection of tools for the automated retrieval and integration of protein sequences and structures from different source databases and their subsequent integration into a local data warehouse system. The initial step in the construction of the database consisted of the selection of seed sequences of 62 proteins which represent members of the different ThDP-dependent protein families (Table A1, Additional file 1). Seed sequences were selected based on the enzymatic activity of the protein and the structural arrangement of protein domains. This selection was based on previous work [17,18] which divided the members of the ThDP-dependent enzymes in different protein families.

### Database establishment

**Figure 1:**
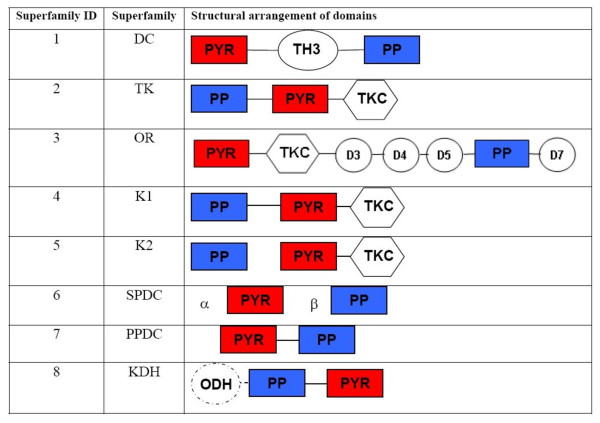
**Structural arrangement of protein domains of the superfamilies of the TEED**. All protein families are listed with their internal superfamily ID, the superfamily name and a 2D representation of the domain arrangement.

The combination of previous classification schemes resulted in 8 different superfamilies, DC (decarboxylase), TK (transketolase), OR (oxidoreductase), and two subfamilies K1 and K2 of the KD (2-ketoacid dehydrogenase) family. In addition to these families, the SPDC (sulfopyruvate decarboxylase), the PPDC (phosphopyruvate decarboxylase), and the KDH (α-ketoglutarate dehydrogenase) family were included (Figure 1). To populate the TEED, a BLAST search against the sequence database at NCBI http://www.ncbi.nlm.nih.gov was carried out for each seed sequence with an E-value cut off of 10^-5^. New protein entries were assigned to a homologous protein family based on their sequence similarity to one of the seed sequences. If the sequence similarity was less than 60%, a protein was assigned to a new homologous family. The families were subsequently manually evaluated and adjusted: protein fragments were merged into the respective homologous family, and proteins with high sequence similarity but a different domain organization were separated into different protein families. This resulted in 63 different homologous families.

Sequence entries with more than 98% sequence identity which shared the same source organism were assigned to the same protein entry. If more than one sequence was assigned to the same protein, the longest sequence was set as the reference sequence. If structural information was available for protein entries, structural monomers were downloaded from the Protein Data Bank [25] and stored as structure entries. Secondary structure information was calculated by DSSP [26] and displayed in the annotated multisequence alignments which were generated by ClustalW (v1.83) with default parameters [27]. Additional annotation on structurally or functionally relevant residues (active site, disulfide bridges, signal peptide) were extracted from the NCBI entry and the respective residues were annotated in the TEED. Abbreviations for the established protein families are available in tabular form (Table A1, Additional file 1).

### Features and functionalities

The online version of the TEED offers pre-calculated multisequence alignments and can be browsed by families, organisms, or structures. Phylogenetic trees are visualized by the program PHYLODENDRON [28]. The PP and PYR domain of each ThDP-dependent protein family was manually annotated. If structural information for a protein homologous family was available, a structural alignment of the available structures was performed using STAMP [29].

**Figure 2:**
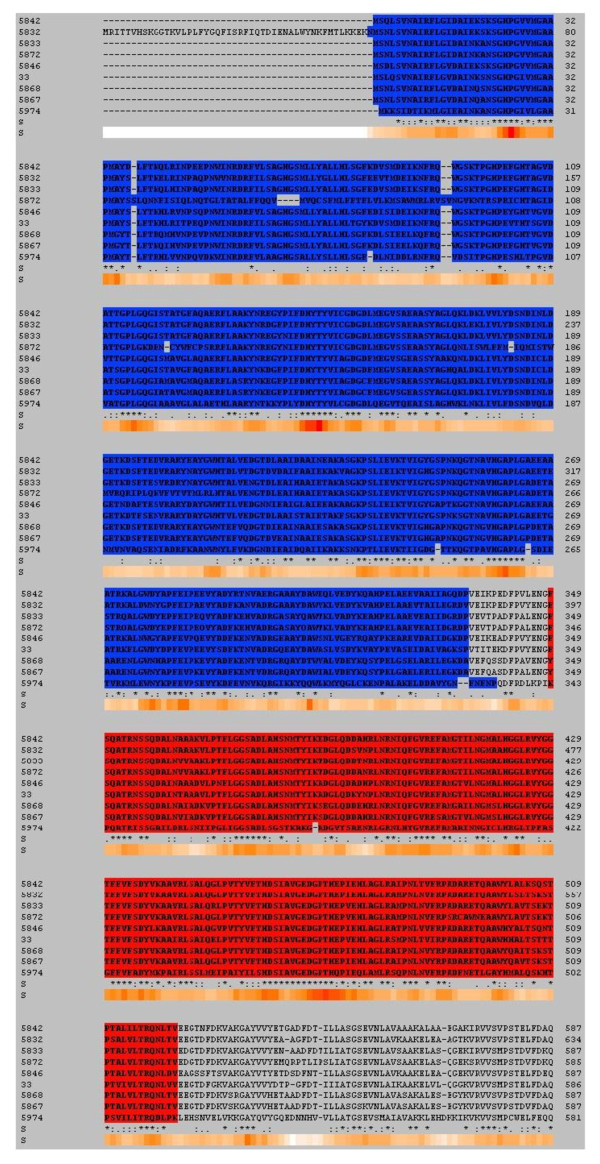
**Multisequence alignment of ThDP dependent proteins with annotated domains**. The pyrophosphate (PP) domain is coloured in blue, the pyrimidine (PYR) domain is coloured red. Annotated PP and PYR domains are available for all protein families in the TEED. The displayed multisequence alignment is taken from the transketolase homologous protein family (TEED ID 33)

If no structure information was available, a set of sequences from the respective homologous protein family was selected and used to create a multisequence alignment. A reliable alignment was ensured by performing this analysis for each homologous family separately to ensure a high degree of sequence similarity. This set consisted of full length sequences from different organisms, excluding protein fragments. The information on the domain boundaries for these sequences was retrieved from InterProScan [30]. Since in many cases, information on the exact N- and C- terminal boundaries for each domain was inconsistent, the boundaries were preferably assigned in well conserved regions rather than in more variable regions. For each multisequence alignment, a Hidden Markov Model (HMM) was created using HMMER [31]. For each homologous family, the individual HMM was used to perform alignments of every protein sequence of this family against the annotated multisequence alignment. Based on this alignment, the PP and PYR domain annotations from the annotated sequences were transferred to every sequence (Figure 2). Annotation information of the PP and PYR domains is displayed for each pre-calculated alignment of homologous families or superfamilies and allows the systematic analysis and evaluation of properties and relationships of these domains. The TEED consists of 12048 sequence entries which were assigned to 9443 different proteins and 379 structure entries. The largest superfamily is the DC family. It consists of more than 4000 sequence entries and accounts for 35% of all sequence entries. The TK and OR families are of comparable size (2600 and 2257 sequence entries, respectively) and account for 21% and 19%, respectively. The source organism of the majority of ThDP-dependent enzymes in the TEED are bacteria (87%).

### Human ThDP-dependent enzymes

66 sequence entries from the TEED are of human origin (excluding sequences from crystal structure chains). Due to their medical importance they were systematically analysed. All human ThDP-dependent enzymes belong to only three superfamilies, the DC, TK, and K2 superfamily (Table A2, Additional file 2). The 66 sequences belong to 20 different proteins with several isoforms. The transketolase (gi: 205277463) with most isoforms (12) is implicated in the latent genetic disease Wernicke-Korsakoff syndrome [32] and has been found to be differentially expressed in the dorsolateral prefrontal cortex from patients with schizophrenia. Another human ThDP-dependent enzyme with many isoforms (7) is the 2-oxoisovalerate dehydrogenase subunit alpha (gi: 548403), also known as branched-chain alpha-keto acid dehydrogenase. This protein is involved in the catabolism of amino acids like isoleucine, leucine, and valine, and a defect causes the accumulation of these amino acids which leads to the maple syrup urine disease [33]. One third of all sequence entries was labelled as 'putative' or 'unnamed' in the GenBank, and was assigned to a specific protein or protein family based on sequence similarity (Table A2, Additional file 2). However, because the function and substrate specificity can vary considerably even between homologous proteins, the assignment of a biochemical property based on sequence similarity only should be regarded as putative. All sequence entries were compared to the respective full sequence and were subsequently classified as either fragments or SNPs. Fragments consist of parts of the full sequence but show no exchange of amino acids while SNPs always show an exchange of amino acids (Table A2, Additional file 2).

## Utility and discussion

The analysis of the human ThDP-dependent enzymes led to a reliable classification of several, previously unclassified proteins and demonstrates the advantage of a highly enriched database of a specific protein family. SNPs have been shown to play an important role in tumor development [34,35] therefore a complete analysis for SNPs was included in the analysis of human ThDP-dependent enzymes. This analysis of SNPs is limited to sequences retrieved from GenBank [36] and thus complements specialised SNP repositories such as the dbSNP [37]. Our analysis demonstrates that GenBank annotations are often incomplete and unreliable for the identification of proteins or protein variants. The transketolase (gi: 205277463) includes 12 different isoforms, of which 6 have been designated as protein fragments. Of these, only one sequence (gi: 193787540) shows an internal deletion, suggesting a truly altered protein product. The other 5 isoforms only show truncated N-termini and therefore could be sequencing artefacts of the original protein.

This kind of analysis is not limited to proteins from a specific organism but can be expanded to cover protein superfamilies or specific homologous families. It has been shown previously that a systematic classification of protein families can be used as a reliable framework for systematic analyses of protein families [38,39] and for the engineering of protein mutants with improved biochemical properties [40,41]. With the implemented domain annotation, an analysis is not limited to the whole protein sequence but protein families can also be specifically analyzed for differences and conserved features in the PP and PYR domains.

### Web accessibility

The database can be accessed on the level of sequence, structure, or organism. All protein entries link to the respective NCBI entries. Annotated multiple sequence alignments and phylogenetic trees are provided via the online accessible version of the TEED at http://www.teed.uni-stuttgart.de. For each family, the level of amino acid conservation is calculated by PLOTCON [42]. BLAST searches [43] can be performed against the TEED using a local BLAST interface. Updates for the TEED will be performed regularly using an automated scripting system. For new sequence entries referring to a new structure in the Protein Data Bank (PDB), structure information is updated as well. New sequence and structure entries are assigned to existing homologous families and superfamilies based on their sequence similarity.

## Conclusions

The Thiamine diphosphate dependent Enzyme Engineering Database (TEED) has been designed to serve as a navigation and analysis tool for the large and diverse family of ThDP-dependent enzymes. The annotation of the conserved pyrophosphate (PP) and pyrimidine (PYR) domains allows for a direct comparison and analysis of these domains between different families. Thus the TEED is a valuable tool for the study of the protein families of ThDP-dependent enzymes.

## Availability and requirements

The Thiamine diphosphate dependent Enzyme Engineering Database (TEED) is online accessible at http://www.teed.uni-stuttgart.de. All information on families, sequence and structure data, as well as alignments and phylogenetic trees can be accessed by manual download.

## List of abbreviations

BLAST: Basic Local Alignment Search Tool; DSSP: Define Secondary Structure of Proteins; DWARF: Data warehouse system for analyzing protein families; HMM: Hidden Markov Model; SNP: Single-nucleotide polymorphism; TEED: Thiamine diphosphate dependent Enzyme Engineering Database; ThDP: Thiamine diphosphate

## Authors' contributions

MW established and annotated the database and wrote the manuscript. RR assisted in the implementation of the database and contributed to writing of the manuscript. JP supervised the project and finalized the manuscript. All authors read and approved the final manuscript.

## Supplementary Material

Additional file 1 **Microsoft Word 2003**. Sequences of ThDP-dependent enzymes which were used to establish the TEED Click here for file

Additional file 2 **Microsoft Word 2003**. Sequences of human ThDP-dependent enzymes Click here for file
