# Supplementary material for: The Thiamine diphosphate dependent Enzyme Engineering Database: A tool for the systematic analysis of sequence and structure relations
Source: BMC Biochem. 2010 Feb 1;11:9. doi: 10.1186/1471-2091-11-9 (PMC2831816; doi:10.1186/1471-2091-11-9)
Supplement: Additional file 1 — Microsoft Word 2003. Sequences of ThDP-dependent enzymes which were used to establish the TEED [file 1471-2091-11-9-S1.DOC]

**The Thiamine diphosphate dependent Enzyme Engineering Database: A tool for the systematic analysis of sequence and structure relations**

**Michael Widmann,^1^ Robert Radloff, ^1^ and Jürgen Pleiss ^1§^**

^1^ Institute of Technical Biochemistry, University of Stuttgart,

Allmandring 31, 70569 Stuttgart, Germany

**Additional file 1**

**Table A1. Sequences of ThDP-dependent enzymes which were used to establish the TEED.** Sequences are annotated with accession numbers, organism of origin and references.

| **Family** | **Accession number (gi)** | | ***Organism*** | **EC** | **Reference** |
| --- | --- | --- | --- | --- | --- |
| ***DC Superfamily*** | | | | | |
|  |  | |  |  |  |
| POX | 29337215 | | ***Lactobacillus plantarum*** | 1.2.3.3 | [1] |
| POX (Cyt.) | 130693 | | ***Escherichia coli*** | 1.2.2.2 | [2] |
| IPDC | 118333 | | ***Enterobacter cloacae*** | 4.1.1.74 | [3, 4] |
| PhePDC | 6320588 | | ***Saccharomyces cerevisiae*** | 4.1.1.- | [5] |
| PDC | 515237 | | ***Saccharomyces cerevisiae*** | 4.1.1.1 | [6, 7] |
|  | 118391 | | ***Zymomonas mobilis*** | 4.1.1.1 | [8] |
| BFDC | 3915757 | | ***Pseudomonas putida*** | 4.1.1.7 | [9, 10] |
| OCDC | 730220 | | ***Oxalobacter formigenes*** | 4.1.1.8 | [11] |
| AHAS | 124373 | | ***Escherichia coli*** | 2.2.1.6 | [12] |
|  | 33112641 | | ***Escherichia coli*** | 2.2.1.6 | [12] |
|  | 2507470 | | ***Escherichia coli*** | 2.2.1.6 | [12] |
|  | 124376 | | ***Saccharomyces cerevisiae*** | 2.2.1.6 | [13] |
|  | 124374 | | ***Klebsiella pneumoniae*** | 2.2.1.6 | [14] |
|  | 75172476 | | ***Lolium multiflorum*** | 2.2.1.6 |  |
| BAL | 1705519 | | ***Pseudomonas fluorescens*** | 4.1.2.38 | [15, 16] |
| CEAS | 75488972 | | ***Streptomyces clavuligerus*** | - | [17] |
| GXC | 84028422 | | ***Escherichia coli*** | 4.1.1.47 | [18] |
| CDP-ADS | unpublished | | ***Yersinia pseudotuberculosis*** |  | [19, 20] |
| kdcA | 75369656 | | ***Lactococcus lactis*** |  | [21] |
| SAAT | 39932465 | | ***Desulfonispora thiosulfatigenes*** | 2.3.3.15 | [22] |
| 2-HPCL | 20455027 | | ***Homo sapiens*** |  | [23, 24] |
|  | 75174050 | | ***Arabidopsis thaliana*** |  |  |
| SEPHCHC (MenD) | 2507472 | | ***Escherichia coli*** | 2.2.1.9 | [25, 26] |
|  | 15790176 | | ***Halobacterium sp.*** | 2.2.1.9 | [26] |
|  | 12323219 | | ***Arabidopsis thaliana*** | 2.2.1.9 | [26] |
| CDH | 185177534 | | ***Azoarcus sp.*** |  | [27] |
| THcHDOH | 81687921 | | ***Bacillus cereus*** | 3.7.1.n2 | [28] |
| pigD | 75361841 | | ***Serratia marcescens*** |  | [29] |
| ***TK Superfamily*** | | | | | |
|  | |  |  |  |  |
| TK | | 1351256 | ***Saccharomyces cerevisiae*** | 2.2.1.1 | [30] |
|  | | 1729976 | ***Homo sapiens*** | 2.2.1.1 | [31] |
|  | | 54042066 | ***Escherichia coli*** | 2.2.1.1 | [32] |
|  | | 169834000 | ***Streptococcus pneumoniae*** | 2.2.1.1 | [33] |
| PK | | 15214330 | ***Bifidobacterium animalis*** | 4.1.2.9 | [34] |
|  | | 21363093 | ***Lactobacillus pentosus*** | 4.1.2.9 | [35] |
| DHAS | | 108936021 | ***Pichia angusta*** | 2.2.1.3 | [36] |
| DXPS | | 2501357 | ***Escherichia coli*** | 2.2.1.7 | [37] |
|  | | 81479889 | ***Fusobacterium nucleatum*** | 4.2.1.- | [38] |
|  | | 122989637 | ***Candidatus Kuenenia stuttgartiensis*** | 2.2.1.7 | [39] |
|  | |  |  |  |  |
| ***OR Superfamily*** | | | | | |
|  | |  |  |  |  |
| PFOR | | 75499539 | ***Desulfovibrio africanus*** |  | [40, 41] |
| PFOR α | | 6685746 | ***Methanobacterium thermoautotrophicum*** | 1.2.7.1 | [42] |
| PFOR β | | 6685734 |  | 1.2.7.1 | [42] |
| PFOR γ | | 6685735 |  | 1.2.7.1 | [42] |
| PFOR δ | | 6685747 |  | 1.2.7.1 | [42] |
| KOR α | | 6685587 | ***Archaeoglobus fulgidus*** | 1.2.7.3 | [43] |
| KOR β | | 6685588 |  | 1.2.7.3 | [43] |
| KOR γ | | 6685586 |  | 1.2.7.3 | [43] |
| KOR δ | | 74570221 |  | 1.2.7.3 | [43] |
| VOR α | | 6686058 | ***Pyrococcus horikoshii*** | 1.2.7.7 | [44] |
| VOR β | | 6686093 |  | 1.2.7.7 | [44] |
| VOR γ | | 6685737 |  | 1.2.7.7 | [44] |
| VOR δ | | 6686092 |  | 1.2.7.7 | [44] |
| IOR α | | 62296914 | ***Pyrococcus kodakaraensis*** | 1.2.7.8 | [45] |
| IOR β | | 62296919 |  | 1.2.7.8 | [45] |
| ***K1 Superfamily*** | | | | | |
|  | |  |  |  |  |
| PDH | | 84027826 | ***Escherichia coli*** | 1.2.4.1 | [46, 47] |
|  | |  |  |  |  |
| ***K2 Superfamily*** | | | | | |
|  | |  |  |  |  |
| BCDH α | | 548403 | ***Homo sapiens*** | 1.2.4.4 | [48] |
| BCDH β | | 129034 |  | 1.2.4.4 | [48] |
| AODH α | | 113136 | ***Ralstonia eutropha*** | 1.1.1.- | [49] |
| AODH β | | 113137 |  | 1.1.1.- | [49] |
|  | |  |  |  |  |
| ***SPDC Superfamily*** | | | | | |
|  | |  |  |  |  |
| SPDC α | | 17432993 | ***Methanocaldococcus jannaschii*** | 4.1.1.79 | [50] |
| SPDC β | | 17432994 |  | 4.1.1.79 | [50] |
|  | |  |  |  |  |
| ***PPDC Superfamily*** | | | | | |
|  | |  |  |  |  |
| PPDC | | 22654224 | ***Streptomyces hygroscopicus*** | 4.1.1.82 | [51] |
|  | |  |  |  |  |
| ***KDH Superfamily*** | | | | | |
|  | |  |  |  |  |
| **OGDC** | | 160395583 | ***Mycobacterium tuberculosis*** | 4.1.1.71 | [52, 53] |
|  | |  |  |  |  |

**DC**

**2-HPCL** 2-hydroxyphytanoyl-CoA lyase, 2-hydroxyacyl-CoA lyase; **AHAS** acetohydroxyacid synthase; **BAL** benzaldehyde aldolase; **BFDC** benzoylformate decarboxylase; **CDH** cyclohexane- 1,2-dione hydrolase; **CDP-ADS (YerE)** CDP-4-aceto-3,6-dideoxygalactose synthase (YerE ); **CEAS** N2-(2-carboxyethyl) arginine synthase; **GXC** glyoxylate carboligase; **IPDC** indolepyruvate decarboxylase; **OCDC** oxalyl-CoA decarboxylase; **PDC** pyruvate decarboxylase; **PhePDC** phenylpyruvate decarboxylase; **POX** pyruvate oxidase; **POX (Cyt)** pyruvate dehydrogenase [cytochrome]; **SAAT** sulfoacetaldehyde acetyltransferase; **SEPHCHC** 2-succinyl-5-enolpyruvyl-6-hydroxy-3-cyclohexene-1-carboxylic-acid synthase; **THcHDOH** 3D-(3,5/4)-trihydroxycyclohexane-1,2-dione hydrolase; **pigD** protein pigD

**TK**

**DHAS** dihydroxyacetone synthase; **DXPS** 1-deoxy-D-xylulose-5-phosphate synthase; **PK** phosphoketolase (xylulose-5-phosphate/fructose-6-phosphate); **TK** transketolase

**OR**

**IOR** indolepyruvate ferredoxin oxidoreductase; **KOR** 2-keto(oxo)glutarate ferredoxin oxidoreductase; **PFOR** pyruvate ferredoxin oxidoreductase; **VOR** 2-keto(oxo)isovalerate ferredoxin oxidoreductase

**K1**

**PDH** pyruvate dehydrogenase E1 component

**K2**

**BCDH** branched-chain 2-ketoacid dehydrogenase; **AODH** acetoin dehydrogenase;

**SPDC**

**SPDC** sulfopyruvate decarboxylase

**PPDC**

**PPDC** phosphonopyruvate decarboxylase

**KDH**

**OGDC** 2-oxo(keto)glutarate decarboxylase, **OGDH** 2-oxoglutarate dehydrogenase

1. Muller YA, Schumacher G, Rudolph R, Schulz GE: **The refined structures of a stabilized mutant and of wild-type pyruvate oxidase from Lactobacillus plantarum**. *J Mol Biol* 1994, **237**(3):315-335.

2. Neumann P, Weidner A, Pech A, Stubbs MT, Tittmann K: **Structural basis for membrane binding and catalytic activation of the peripheral membrane enzyme pyruvate oxidase from Escherichia coli**. *Proc Natl Acad Sci U S A* 2008, **105**(45):17390-17395.

3. Schutz A, Sandalova T, Ricagno S, Hubner G, Konig S, Schneider G: **Crystal structure of thiamindiphosphate-dependent indolepyruvate decarboxylase from Enterobacter cloacae, an enzyme involved in the biosynthesis of the plant hormone indole-3-acetic acid**. *Eur J Biochem* 2003, **270**(10):2312-2321.

4. Schutz A, Golbik R, Tittmann K, Svergun DI, Koch MH, Hubner G, Konig S: **Studies on structure-function relationships of indolepyruvate decarboxylase from Enterobacter cloacae, a key enzyme of the indole acetic acid pathway**. *Eur J Biochem* 2003, **270**(10):2322-2331.

5. Vuralhan Z, Morais MA, Tai SL, Piper MD, Pronk JT: **Identification and characterization of phenylpyruvate decarboxylase genes in Saccharomyces cerevisiae**. *Appl Environ Microbiol* 2003, **69**(8):4534-4541.

6. Dyda F, Furey W, Swaminathan S, Sax M, Farrenkopf B, Jordan F: **Catalytic centers in the thiamin diphosphate dependent enzyme pyruvate decarboxylase at 2.4-A resolution**. *Biochemistry* 1993, **32**(24):6165-6170.

7. Rosche B, Breuer M, Hauer B, Rogers PL: **Screening of yeasts for cell-free production of (R)-phenylacetylcarbinol**. *Biotechnol Lett* 2003, **25**(11):841-845.

8. Dobritzsch D, Konig S, Schneider G, Lu G: **High resolution crystal structure of pyruvate decarboxylase from Zymomonas mobilis. Implications for substrate activation in pyruvate decarboxylases**. *J Biol Chem* 1998, **273**(32):20196-20204.

9. Hasson MS, Muscate A, McLeish MJ, Polovnikova LS, Gerlt JA, Kenyon GL, Petsko GA, Ringe D: **The crystal structure of benzoylformate decarboxylase at 1.6 A resolution: diversity of catalytic residues in thiamin diphosphate-dependent enzymes**. *Biochemistry* 1998, **37**(28):9918-9930.

10. Lingen B, Kolter-Jung D, Dunkelmann P, Feldmann R, Grotzinger J, Pohl M, Muller M: **Alteration of the substrate specificity of benzoylformate decarboxylase from Pseudomonas putida by directed evolution**. *Chembiochem* 2003, **4**(8):721-726.

11. Berthold CL, Moussatche P, Richards NG, Lindqvist Y: **Structural basis for activation of the thiamin diphosphate-dependent enzyme oxalyl-CoA decarboxylase by adenosine diphosphate**. *J Biol Chem* 2005, **280**(50):41645-41654.

12. Engel S, Vyazmensky M, Geresh S, Barak Z, Chipman DM: **Acetohydroxyacid synthase: a new enzyme for chiral synthesis of R-phenylacetylcarbinol**. *Biotechnol Bioeng* 2003, **83**(7):833-840.

13. Pang SS, Duggleby RG, Guddat LW: **Crystal structure of yeast acetohydroxyacid synthase: a target for herbicidal inhibitors**. *J Mol Biol* 2002, **317**(2):249-262.

14. Pang SS, Duggleby RG, Schowen RL, Guddat LW: **The crystal structures of Klebsiella pneumoniae acetolactate synthase with enzyme-bound cofactor and with an unusual intermediate**. *J Biol Chem* 2004, **279**(3):2242-2253.

15. Demir AS, Sesenoglu O, Dunkelmann P, Muller M: **Benzaldehyde lyase-catalyzed enantioselective carboligation of aromatic aldehydes with mono- and dimethoxy acetaldehyde**. *Org Lett* 2003, **5**(12):2047-2050.

16. Maraite A, Schmidt T, Ansorge-Schumacher MB, Brzozowski AM, Grogan G: **Structure of the ThDP-dependent enzyme benzaldehyde lyase refined to 1.65 A resolution**. *Acta Crystallogr Sect F Struct Biol Cryst Commun* 2007, **63**(Pt 7):546-548.

17. Caines ME, Elkins JM, Hewitson KS, Schofield CJ: **Crystal structure and mechanistic implications of N2-(2-carboxyethyl)arginine synthase, the first enzyme in the clavulanic acid biosynthesis pathway**. *J Biol Chem* 2004, **279**(7):5685-5692.

18. Kaplun A, Binshtein E, Vyazmensky M, Steinmetz A, Barak Z, Chipman DM, Tittmann K, Shaanan B: **Glyoxylate carboligase lacks the canonical active site glutamate of thiamine-dependent enzymes**. *Nature Chemical Biology* 2008, **4**(2):113-118.

19. Chen HW, Guo ZH, Liu HW: **Biosynthesis of yersiniose: Attachment of the two-carbon branched-chain is catalyzed by a thiamine pyrophosphate-dependent flavoprotein**. *Journal of the American Chemical Society* 1998, **120**(45):11796-11797.

20. Mansoorabadi SO, Thibodeaux CJ, Liu HW: **The diverse roles of flavin coenzymes--nature's most versatile thespians**. *J Org Chem* 2007, **72**(17):6329-6342.

21. Berthold CL, Gocke D, Wood D, Leeper FJ, Pohl M, Schneider G: **Structure of the branched-chain keto acid decarboxylase (KdcA) from Lactococcus lactis provides insights into the structural basis for the chemoselective and enantioselective carboligation reaction**. *Acta Crystallographica Section D-Biological Crystallography* 2007, **63**:1217-1224.

22. Ruff J, Denger K, Cook AM: **Sulphoacetaldehyde acetyltransferase yields acetyl phosphate: purification from Alcaligenes defragrans and gene clusters in taurine degradation**. *Biochem J* 2003, **369**(Pt 2):275-285.

23. Foulon V, Antonenkov VD, Croes K, Waelkens E, Mannaerts GP, Van Veldhoven PP, Casteels M: **Purification, molecular cloning, and expression of 2-hydroxyphytanoyl-CoA lyase, a peroxisomal thiamine pyrophosphate-dependent enzyme that catalyzes the carbon-carbon bond cleavage during alpha-oxidation of 3-methyl-branched fatty acids**. *Proc Natl Acad Sci U S A* 1999, **96**(18):10039-10044.

24. Casteels M, Foulon V, Mannaerts GP, Van Veldhoven PP: **Alpha-oxidation of 3-methyl-substituted fatty acids and its thiamine dependence**. *European Journal of Biochemistry* 2003, **270**(8):1619-1627.

25. Dawson A, Fyfe PK, Hunter WN: **Specificity and reactivity in menaquinone biosynthesis: the structure of Escherichia coli MenD (2-succinyl-5-enolpyruvyl-6-hydroxy-3-cyclohexadiene-1-carboxylate synthase)**. *J Mol Biol* 2008, **384**(5):1353-1368.

26. Bhasin M, Billinsky JL, Palmer DR: **Steady-state kinetics and molecular evolution of Escherichia coli MenD [(1R,6R)-2-succinyl-6-hydroxy-2,4-cyclohexadiene-1-carboxylate synthase], an anomalous thiamin diphosphate-dependent decarboxylase-carboligase**. *Biochemistry* 2003, **42**(46):13496-13504.

27. Harder J: **Anaerobic degradation of cyclohexane-1,2-diol by a new Azoarcus species**. *Arch Microbiol* 1997, **168**(3):199-204.

28. Yoshida K, Yamaguchi M, Morinaga T, Kinehara M, Ikeuchi M, Ashida H, Fujita Y: **myo-Inositol catabolism in Bacillus subtilis**. *J Biol Chem* 2008, **283**(16):10415-10424.

29. Williamson NR, Simonsen HT, Ahmed RA, Goldet G, Slater H, Woodley L, Leeper FJ, Salmond GP: **Biosynthesis of the red antibiotic, prodigiosin, in Serratia: identification of a novel 2-methyl-3-n-amyl-pyrrole (MAP) assembly pathway, definition of the terminal condensing enzyme, and implications for undecylprodigiosin biosynthesis in Streptomyces**. *Mol Microbiol* 2005, **56**(4):971-989.

30. Nikkola M, Lindqvist Y, Schneider G: **Refined structure of transketolase from Saccharomyces cerevisiae at 2.0 A resolution**. *J Mol Biol* 1994, **238**(3):387-404.

31. Obiol-Pardo C, Rubio-Martinez J: **Homology modeling of human transketolase: description of critical sites useful for drug design and study of the cofactor binding mode**. *J Mol Graph Model* 2009, **27**(6):723-734.

32. Asztalos P, Parthier C, Golbik R, Kleinschmidt M, Hubner G, Weiss MS, Friedemann R, Wille G, Tittmann K: **Strain and near attack conformers in enzymic thiamin catalysis: X-ray crystallographic snapshots of bacterial transketolase in covalent complex with donor ketoses xylulose 5-phosphate and fructose 6-phosphate, and in noncovalent complex with acceptor aldose ribose 5-phosphate**. *Biochemistry* 2007, **46**(43):12037-12052.

33. Reizer J, Reizer A, Bairoch A, Saier MH, Jr.: **A diverse transketolase family that includes the RecP protein of Streptococcus pneumoniae, a protein implicated in genetic recombination**. *Res Microbiol* 1993, **144**(5):341-347.

34. Meile L, Rohr LM, Geissman TA, Herensperger M, Teuber M: **Characterization of the D-xylulose 5-phosphate/D-Fructose 6-phosphate phosphoketolase gene (xfp) from Bifidobacterium lactis**. *Journal of Bacteriology* 2001, **183**(9):2929-2936.

35. Posthuma CC, Bader R, Engelmann R, Postma PW, Hengstenberg W, Pouwels PH: **Expression of the xylulose 5-phosphate phosphoketolase gene, xpkA, from Lactobacillus pentosus MD363 is induced by sugars that are fermented via the phosphoketolase pathway and is repressed by glucose mediated by CcpA and the mannose phosphoenolpyruvate phosphotransferase system**. *Applied and Environmental Microbiology* 2002, **68**(2):831-837.

36. Janowicz ZA, Eckart MR, Drewke C, Roggenkamp RO, Hollenberg CP, Maat J, Ledeboer AM, Visser C, Verrips CT: **Cloning and characterization of the DAS gene encoding the major methanol assimilatory enzyme from the methylotrophic yeast hansenula-polymorpha**. *Nucleic Acids Research* 1985, **13**(9):3043-3062.

37. Xiang S, Usunow G, Lange G, Busch M, Tong L: **Crystal structure of 1-deoxy-d-xylulose 5-phosphate synthase, a crucial enzyme for isoprenoids biosynthesis**. *Journal of Biological Chemistry* 2007, **282**(4):2676-2682.

38. Kapatral V, Anderson I, Ivanova N, Reznik G, Los T, Lykidis A, Bhattacharyya A, Bartman A, Gardner W, Grechkin G *et al*: **Genome sequence and analysis of the oral bacterium Fusobacterium nucleatum strain ATCC 25586**. *J Bacteriol* 2002, **184**(7):2005-2018.

39. Strous M, Pelletier E, Mangenot S, Rattei T, Lehner A, Taylor MW, Horn M, Daims H, Bartol-Mavel D, Wincker P *et al*: **Deciphering the evolution and metabolism of an anammox bacterium from a community genome**. *Nature* 2006, **440**(7085):790-794.

40. Chabriere E, Vernede C, Guigliarelli B, Charon MH, Hatchikian EC, Fontecilla-Camps JC: **Crystal structure of the free radical intermediate of pyruvate : ferredoxin oxidoreductase**. *Science* 2001, **294**(5551):2559-2563.

41. Chabriere E, Charon MH, Volbeda A, Pieulle L, Hatchikian EC, Fontecilla-Camps JC: **Crystal structures of the key anaerobic enzyme pyruvate : ferredoxin oxidoreductase, free and in complex with pyruvate**. *Nature Structural Biology* 1999, **6**(2):182-190.

42. Smith DR, DoucetteStamm LA, Deloughery C, Lee HM, Dubois J, Aldredge T, Bashirzadeh R, Blakely D, Cook R, Gilbert K *et al*: **Complete genome sequence of Methanobacterium thermoautotrophicum Delta H: Functional analysis and comparative genomics**. *Journal of Bacteriology* 1997, **179**(22):7135-7155.

43. Klenk HP, Clayton RA, Tomb JF, White O, Nelson KE, Ketchum KA, Dodson RJ, Gwinn M, Hickey EK, Peterson JD *et al*: **The complete genome sequence of the hyperthermophilic, sulphate-reducing archaeon Archaeoglobus fulgidus**. *Nature* 1997, **390**(6658):364-&.

44. Kawarabayasi Y, Sawada M, Horikawa H, Haikawa Y, Hino Y, Yamamoto S, Sekine M, Baba S, Kosugi H, Hosoyama A *et al*: **Complete sequence and gene organization of the genome of a hyper-thermophilic archaebacterium, Pyrococcus horikoshii OT3**. *DNA Res* 1998, **5**(2):55-76.

45. Siddiqui MA, Fujiwara S, Imanaka T: **Indolepyruvate ferredoxin oxidoreductase from Pyrococcus sp. KOD1 possesses a mosaic structure showing features of various oxidoreductases**. *Mol Gen Genet* 1997, **254**(4):433-439.

46. Stephens PE, Darlison MG, Lewis HM, Guest JR: **The pyruvate-dehydrogenase complex of Escherichia-coli-K12 - nucleotide-sequence encoding the pyruvate-dehydrogenase component**. *European Journal of Biochemistry* 1983, **133**(1):155-162.

47. Arjunan P, Chandrasekhar K, Sax M, Brunskill A, Nemeria N, Jordan F, Furey W: **Structural determinants of enzyme binding affinity: The E1 component of pyruvate dehydrogenase from Escherichia coli in complex with the inhibitor thiamin thiazolone diphosphate**. *Biochemistry* 2004, **43**(9):2405-2411.

48. Wynn RM, Kato M, Machius M, Chuang JL, Li J, Tomchick DR, Chuang DT: **Molecular mechanism for regulation of the human mitochondrial branched-chain alpha-ketoacid dehydrogenase complex by phosphorylation**. *Structure* 2004, **12**(12):2185-2196.

49. Priefert H, Hein S, Kruger N, Zeh K, Schmidt B, Steinbuchel A: **Identification and molecular characterization of the Alcaligenes eutrophus H16 aco operon genes involved in acetoin catabolism**. *J Bacteriol* 1991, **173**(13):4056-4071.

50. Graupner M, Xu H, White RH: **Identification of the gene encoding sulfopyruvate decarboxylase, an enzyme involved in biosynthesis of coenzyme M**. *J Bacteriol* 2000, **182**(17):4862-4867.

51. Nakashita H, Kozuka K, Hidaka T, Hara O, Seto H: **Identification and expression of the gene encoding phosphonopyruvate decarboxylase of Streptomyces hygroscopicus**. *Biochim Biophys Acta* 2000, **1490**(1-2):159-162.

52. Tian J, Bryk R, Itoh M, Suematsu M, Nathan C: **Variant tricarboxylic acid cycle in Mycobacterium tuberculosis: identification of alpha-ketoglutarate decarboxylase**. *Proc Natl Acad Sci U S A* 2005, **102**(30):10670-10675.

53. Tian J, Bryk R, Shi S, Erdjument-Bromage H, Tempst P, Nathan C: **Mycobacterium tuberculosis appears to lack alpha-ketoglutarate dehydrogenase and encodes pyruvate dehydrogenase in widely separated genes**. *Mol Microbiol* 2005, **57**(3):859-868.
