# Supplementary material for: The Thiamine diphosphate dependent Enzyme Engineering Database: A tool for the systematic analysis of sequence and structure relations
Source: BMC Biochem. 2010 Feb 1;11:9. doi: 10.1186/1471-2091-11-9 (PMC2831816; doi:10.1186/1471-2091-11-9)
Supplement: Additional file 2 — Microsoft Word 2003. Sequences of human ThDP-dependent enzymes [file 1471-2091-11-9-S2.DOC]

**The Thiamine diphosphate dependent Enzyme Engineering Database: A tool for the systematic analysis of sequence and structure relations**

**Michael Widmann,^1^ Robert Radloff, ^1^ and Jürgen Pleiss ^1§^**

^1^ Institute of Technical Biochemistry, University of Stuttgart,

Allmandring 31, 70569 Stuttgart, Germany

**Additional file 2**

**Table A2. Sequences of human ThDP-dependent enzymes.** Protein descriptions are taken from the protein GenBank (gi) entry. Isoforms of proteins are assigned to the same protein ID. Protein classifications state if the protein is considered a full sequence, a fragment of the full sequence or a SNP of the full sequence. Superfamily and homologous family describe the TEED identifiers, the homologous family id is given in brackets. Protein contains the name of the respective protein with the internal TEED protein id in brackets.

| **Superfamily** | **Homologous Family** | **Protein** | **gi** | **Sequence ID** | **Protein description from GenBank** | **Classification** |
| --- | --- | --- | --- | --- | --- | --- |
| DC | AHAS (11) | acetolactate synthase homolog (1433) | 1730288 | 1886 | acetolactate synthase homolog | Full sequence |
| DC | 2-HPCL (21) | hydroxyacyl-CoA lyase (21) | 20455027 | 21 | isoform CRA_a | Full sequence |
| DC | 2-HPCL (21) |  | 119584656 | 3548 | isoform CRA_b | Fragment |
| DC | 2-HPCL (21) |  | 193787013 | 1875 | isoform CRA_c | Fragment |
| DC | 2-HPCL (21) |  | 6841208 | 1744 | HSPC279 | Fragment |
| DC | 2-HPCL (21) |  | 194378616 | 1777 | unnamed protein product | Fragment |
| DC | 2-HPCL (21) |  | 194378068 | 1821 | unnamed protein product | Fragment |
| DC | 2-HPCL (21) |  | 194376964 | 1770 | unnamed protein product | Fragment |
| DC | 2-HPCL (21) |  | 6273457 | 1745 | 2-hydroxyphytanoyl-CoA lyase | Q447H,R543E |
| DC | 2-HPCL (21) | unnamed (1317) | 34531269 | 1741 | unnamed protein product | Full sequence |
| DC | 2-HPCL (21) | unnamed (1378) | 194387780 | 1819 | unnamed protein product | Full sequence |
|  | | | | | | |
| TK | TK (31) | Transketolase (31) | 205277463 | 31 | isoform 1 | Full sequence |
| TK | TK (31) |  | 193787540 | 5096 | isoform 2 | Fragment |
| TK | TK (31) |  | 194381830 | 5080 | unnamed protein product | Fragment |
| TK | TK (31) |  | 38013966 | 5087 | TKT protein | Fragment |
| TK | TK (31) |  | 31417921 | 5108 | TKT protein | Fragment |
| TK | TK (31) |  | 14250367 | 5149 | TKT protein | Fragment |
| TK | TK (31) |  | 193787037 | 5193 | unnamed protein product | Fragment |
| TK | TK (31) |  | 62898960 | 5062 | transketolase variant | Q367R |
| TK | TK (31) |  | 388891 | 5065 | transketolase | T585K, H586T, L587M |
| TK | TK (31) |  | 37267 | 5066 | transketolase | P426A |
| TK | TK (31) |  | 194373693 | 5067 | unnamed protein product | E374G |
| TK | TK (31) |  | 194373793 | 5119 | unnamed protein product | K145N,I378V |
| TK | TK (31) | Transketolase-like 2 (3748) | 119625243 | 5124 | Transketolase-like 2 | Full sequence |
| TK | TK (31) |  | 16553281 | 5235 | unnamed protein product | Fragment |
| TK | TK (31) |  | 189069449 | 5126 | unnamed protein product | Y148H;I444T |
| TK | TK (31) | Transketolase-like 2 (3748) | 16552972 | 5127 | unnamed protein product | F54L;M406I |
| TK | TK (31) |  | 133777215 | 5128 | Transketolase-like 2 | I302V;P311H |
| TK | TK (31) |  | 148744456 | 5120 | Transketolase-like 2 | Q590H |
| TK | TK (31) | Transketolase-like 1 (3781) | 158257954 | 5162 | isoform a | Full sequence |
| TK | TK (31) |  | 221043878 | 5171 | isoform b | Fragment |
| TK | TK (31) |  | 55666480 | 5184 | isoform c | Fragment |
| TK | TK (31) |  | 221043730 | 5206 | unnamed protein product | Y249C;H396Y |
| TK | TK (31) |  | 158257880 | 5164 | unnamed protein product | D26N |
| TK | TK (31) |  | 34190015 | 5165 | Transketolase-like 1 | L24F;I152T |
| TK | TK (31) | Transketolase-like 1 (3791) | 119593156 | 5178 | transketolase-like 1, isoform CRA_b | Full sequence |
| TK | TK (31) | Transketolase-like 1 (3809) | 119593155 | 5204 | transketolase-like 1, isoform CRA_a | Full sequence |
| TK | TK (31) |  | 119593159 | 5209 | transketolase-like 1, isoform CRA_e | Fragment |
| TK | TK (31) | Transketolase-like 1 (3810) | 119593157 | 5205 | transketolase-like 1, isoform CRA_c | Full sequence |
| TK | TK (31) | Transketolase (3797) | 1232175 | 5189 | transketolase | Full sequence |
| TK | TK (31) | Transketolase-like 1 (3855) | 122891454 | 5264 | transketolase-like 1 | Full sequence |
|  | | | | | | |
| K2 | BCDH alpha (56) | 2-oxoisovalerate dehydrogenase subunit alpha (56) | 119577444 | 56 | 2-oxoisovalerate dehydrogenase subunit alpha | Full sequence |
| K2 | BCDH alpha (56) |  | 62089242 | 10805 | branched chain keto acid dehydrogenase E1, alpha polypeptide variant | ΔR287 |
| K2 | BCDH alpha (56) |  | 5705948 | 10841 | branched-chain alpha-keto acid dehydrogenase complex E1 alpha subunit | V1G |
| K2 | BCDH alpha (56) |  | 189055345 | 10802 | unnamed protein product | E377K |
| K2 | BCDH alpha (56) |  | 8176547 | 10803 | branched-chain alpha-keto acid dehydrogenase E1 alpha subunit | V1G |
| K2 | BCDH alpha (56) |  | 386841 | 10806 | branched-chain alpha-keto acid dehydrogenase | S35A |
| K2 | BCDH alpha (56) | 2-oxoisovalerate dehydrogenase subunit alpha (56) | 179360 | 10843 | branched-chain alpha-keto acid dehydrogenase E1-alpha subunit | A181D |
| K2 | BCDH alpha (56) | unnamed (8558) | 194389886 | 10813 | unnamed protein product | Full sequence |
| K2 | BCDH alpha (56) | unnamed (8559) | 34534581 | 10815 | unnamed protein product | Full sequence |
| K2 | BCDH alpha (56) | hypothetical (8572) | 52545799 | 10850 | hypothetical protein | Full sequence |
| K2 |  |  |  |  |  |  |
| K2 | BCDH beta (57) | 2-oxoisovalerate dehydrogenase subunit beta (57) | 129034 | 57 | 2-oxoisovalerate dehydrogenase subunit beta | Full sequence |
| K2 | BCDH beta (57) |  | 221040270 | 11328 | unnamed protein product | Fragment |
| K2 | BCDH beta (57) |  | 119569083 | 11439 | branched chain keto acid dehydrogenase E1, beta polypeptide | Fragment |
| K2 | BCDH beta (57) |  | 747713 | 11309 | unnamed protein product | T303S |
| K2 | BCDH beta (57) |  | 194385640 | 11329 | unnamed protein product | Q276L |
| K2 |  |  |  |  |  |  |
| K2 | AODH alpha (61) | mitochondrial PDHA1 (9896) | 148357460 | 12435 | mitochondrial PDHA1 | Full sequence |
| K2 | AODH alpha (61) |  | 4505685 | 12453 | pyruvate dehydrogenase (lipoamide) alpha 1 precursor | Fragment |
| K2 | AODH alpha (61) |  | 221041292 | 12530 | unnamed protein product | Fragment |
| K2 | AODH alpha (61) |  | 62897039 | 12439 | pyruvate dehydrogenase (lipoamide) alpha 1 variant | M282L |
| K2 | AODH alpha (61) |  | 62897537 | 12440 | pyruvate dehydrogenase (lipoamide) alpha 1 variant | M282L;N328S |
| K2 | AODH alpha (61) |  | 189053388 | 12490 | unnamed protein product | G278E |
| K2 | AODH alpha (61) | pyruvate dehydrogenase E1-alpha precursor (9917) | 387011 | 12463 | pyruvate dehydrogenase E1-alpha precursor | Full sequence |
| K2 | AODH alpha (61) | pyruvate dehydrogenase (9985) | 119626468 | 12556 | hCG1643458 | Full sequence |
| K2 | AODH alpha (61) |  | 66267554 | 12549 | PDHA2 protein | Fragment |
|  | AODH alpha (61) |  | 4885543 | 12546 | pyruvate dehydrogenase (lipoamide) alpha 2 | Fragment |
